# Health systems science and systems thinking: qualitative evaluation of preclinical medical student reflections in a role-play simulation game

**DOI:** 10.1186/s12909-025-07594-w

**Published:** 2025-07-18

**Authors:** Thi Kim Chi Dang, Kyung Hye Park, Sangmi Teresa Lee

**Affiliations:** Department of Medical Education, Yonsei University Wonju College of Medicine, 20 Ilsan-ro, Gangwon-do 26426 Wonju, Republic of Korea

**Keywords:** Simulation training, Experiential learning, Qualitative research, Systems theory, Medical students

## Abstract

**Background:**

The importance of systems thinking in Health Systems Science is increasingly recognised, yet its integration into undergraduate medical education remains inconsistent. Evidence on the use of simulation games to promote experiential learning and systems thinking development during the preclinical phase is also limited. This study examined the effect of the simulation game Friday Night at the ER (FNER) on the development of systems thinking in preclinical medical students. The research questions focused on (1) What core systems thinking strategies affected team performance scores in the FNER game? (2) How were these strategies related to the Habits of a Systems Thinker framework? (3) What emotions and insights did students gain through the FNER game?

**Methods:**

Following simulation-based experiential learning in a systems-focused curriculum, we analysed open-ended reflections from 22 preclinical medical students (mean age 24.3; 11 females, 11 males) using a hybrid qualitative content analysis. Strategy-related responses were examined through directed content analysis informed by two frameworks: core systems thinking strategies embedded in FNER and the Habits of a Systems Thinker, representing foundational Health Systems Science competencies. Emotional and insight-related reflections were analysed inductively to identify effective emergent themes.

**Results:**

Students employed three key strategies—collaboration, data-driven decision-making, and innovation—during gameplay. High-performing teams demonstrated a more frequent and integrated use of these strategies, implicitly applying multiple habits of a systems thinker, such as Habit 1 (seeing the big picture), Habit 6 (changing perspectives), and Habit 11 (considering consequences). Low-performing teams, focused on individual tasks, failed to drive collaboration at the team and organisational levels, and remained stuck in linear thinking. Five effective themes emerged from students’ reflections: collaboration and teamwork, value-driven decision-making, innovation and change, patient-centred care, and indirect experience of hospital systems.

**Conclusions:**

FNER is an educational tool that promotes the use of role-play simulation games to teach systems thinking, a core linking domain in Health Systems Science. By fostering collaboration, innovation, and data-driven decision-making, the game helps preclinical medical students begin forming a professional identity as system-based practice physicians capable of navigating and improving complex healthcare systems.

**Trial registration:**

Not applicable.

## Background

Since the Flexner Report in 1910, medical education has been structured around two core pillars: basic and clinical sciences [1–3]. However, as healthcare systems worldwide face persistent challenges—such as rising costs, suboptimal quality, and increasing medical errors—topics once marginalised or taught as electives (e.g., teamwork, patient-centred care, health policy, quality improvement, and systems thinking) have gained renewed importance [1, 4–6].

Despite the presence of integrative frameworks, such as the Triple Aim and Quadruple Aim, which emphasise improved patient care, population health, and cost reduction, the U.S. healthcare system continues to struggle with inefficiencies and uneven quality [7–11]. In response, medical educators have proposed Health Systems Science (HSS) as a “third pillar” of medical education, complementing both basic and clinical sciences [1, 6, 12, 13]. Since the COVID-19 pandemic and the 2020 revision of the WFME Global Standards, interest in developing and integrating HSS curricula has grown worldwide [14–17].

Among the HSS domains, Systems Thinking (ST) serves as a critical linking domain, connecting both functional (e.g., healthcare policy, informatics) and foundational (e.g., leadership, ethics) domains [18–20]. ST encourages a holistic understanding of the relationships among components within complex systems, such as care teams, hospitals, and broader health systems, and how those components interact dynamically over time [20–22]. It is recognised as a vital competency for enhancing patient safety, promoting interprofessional collaboration, and navigating real-world complexity [20, 23–25].

Despite its growing relevance, ST remains inconsistently integrated into health professions education, and a persistent gap exists between conceptual knowledge and practical application [25–28]. Student engagement is another significant barrier to the adoption of HSS. While some students recognise its relevance, others perceive the content as redundant or lacking direct clinical utility, often comparing it to “eating broccoli” [29–34]. These attitudes highlight the need for innovative, learner-centred approaches to HSS instruction, especially in the preclinical years of medical education.

Simulation games have emerged as a promising method to address this gap. Friday Night at the ER (FNER) has been recommended for teaching ST due to its effectiveness in fostering teamwork, adaptive leadership, and system-level awareness [35–40]. In South Korea, interest in HSS has increased since the pandemic. The Korean Association of Medical Colleges conducted a study on HSS with government funding, introducing FNER as a core educational method for systems thinking and running a pilot course at one university [41–47].

A distinct strength of FNER is its ability to engage students in experiential learning that aligns with the 14 Habits of a Systems Thinker. This framework articulates how systems thinkers observe, analyse, and act in complex systems [20, 48]. These habits include pursuing the big picture, recognising change over time, identifying causal relationships, externalising mental models, and predicting both short-term and long-term outcomes. These habits are foundational to fostering systems thinking in medical learners and are increasingly recognised as essential to HSS competencies [20, 48–50].

Despite the growing use of FNER, most FNER studies focus on nursing or interprofessional students, often emphasising quantitative outcomes. There is a paucity of research on how FNER supports undergraduate medical students’ reflective learning and ST development in preclinical education [35–40, 49].

Therefore, this study aimed to qualitatively explore how preclinical medical students perceive and reflect on their learning experiences after participating in the FNER simulation. This study addressed the following research questions: (1) What core ST strategies affected team performance scores in the FNER game? (2) How were these strategies related to the Habits of a Systems Thinker framework? (3) What emotions and insights did students gain through the FNER game?

## Methods

### Participants

This qualitative study explored the experiential learning of ST among preclinical medical students using the FNER simulation game. The study was embedded in the elective course “Leadership and Systems Thinking,” conducted in October 2023 at Yonsei University Wonju College of Medicine. This course was designed to introduce third-year medical students to core leadership competencies and ST principles through experiential learning. Ethical approval was granted by the Ethics Committee of Yonsei University Wonju Christian Severance Hospital (approval no. CR 2024-0134-001). Given the retrospective nature of the study, the Committee waived the requirement for written informed consent.

A total of 22 third-year students from a six-year medical program participated in the study. These students had not yet entered clinical clerkships and were primarily engaged in integrated basic clinical sciences. The sample comprised 11 female and 11 male students, with a mean age of 24.3 years (range: 24–27 years). All participants submitted written reflections in response to open-ended questions following the simulation gameplay.

### Study design and simulation game

**Fig. 1:**
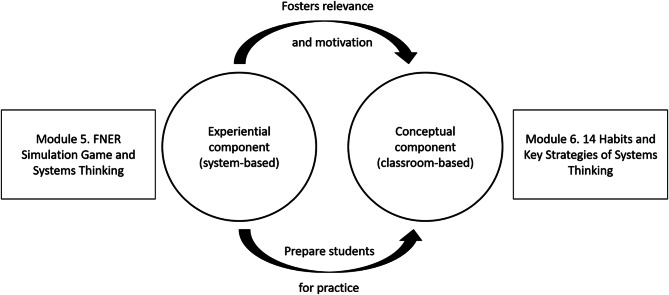
Model for a systems curriculum introducing Systems Thinking to preclinical students

To introduce medical students to the concept of ST and the habits and strategies of a systems thinker within the context of HSS, we employed a systems curriculum model consisting of two pedagogical components: a conceptual, classroom-based module and a system-based, experiential module [1]. These corresponded to Modules 5 and 6 of the courses. In Module 5, students participated in a three-hour FNER simulation session, where they managed a virtual hospital in teams, assuming leadership roles for various units (emergency, surgery, step-down, and critical care) (Fig. 1).

**Fig. 2:**
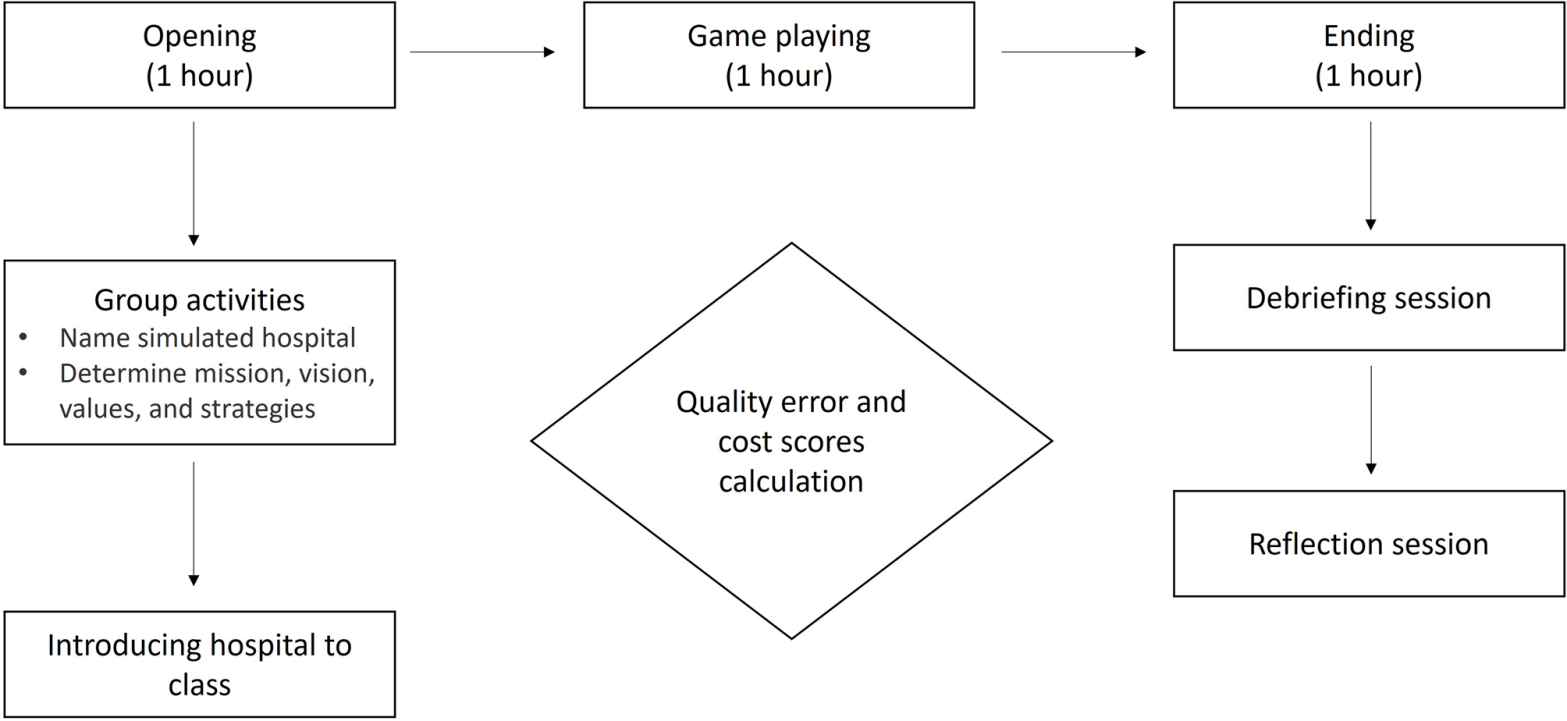
Instructional flow of the FNER simulation class

During the simulation, participants’ performance was assessed using quality and cost scores, which reflected the impact of specific operational decisions made across different hospital departments. Actions such as ambulance diversions, patient wait times, pending service requests, deployment of additional staff, and failure to complete the 24-hour shift influenced these scores to varying degrees, depending on whether they occurred in the emergency department or other units such as surgery, critical care, or step-down wards. Each action carried distinct implications for quality loss and financial cost, prompting participants to weigh trade-offs between efficiency, resource allocation, and patient outcomes. The scoring criteria were made available throughout the simulation via a printed worksheet placed on each team’s table. This worksheet functioned as a passive reference tool that participants could consult at their discretion during gameplay; facilitators did not provide explicit instructions or guidance on its use. The FNER simulation game (available at https://fridaynightattheer.com/), span-ning three hours, was conducted during the fifth week of the course. The instructor completed an online Facilitator course to deliver the course. The key learning outcome of the FNER gameplay module was to promote the core strategies of ST, including collaboration, innovation, and data-driven decision-making. The class was structured as follows (Fig. 2):

In the first hour, students engaged in group activities, where they named their hospital, determined its mission, vision, values, and strategy, and then introduced it to the class. The second hour was spent playing the simulation game. Four participants assumed the role of leader or manager for each of the four units in a healthcare setting: the emergency, surgery, step-down, and critical care units. The fifth learner checked each hour to determine whether the leaders of each unit had recorded the charts correctly. This recorded information was used to calculate the learner group scores in terms of quality and cost outcomes at the end of the game [39]. The game cards determined new patient arrivals, ‘ready to exit’ indicators for patients, and various events that simulate real-life scenarios, such as a doctor being late, a room being renovated, and a staff member going home sick. These situations required the players to react and manage their resources effectively. Learners tracked the data for each department on paperwork forms during the game. The recorded metrics included the number of patients transferred to other hospitals by ambulance, those waiting in each department, and the number of additional personnel hired. It was presented as a bar graph, demonstrating the quality of service they delivered and their financial performance. The team performance score was calculated using the following formula:

Team Performance = (Quality Errors Score + Cost Score)/2.

In this formula, a lower team performance score indicates better overall performance, with fewer quality errors and more efficient cost management, and vice versa. Team scores enable players to relate their behaviours to their performance and provide benchmarks.

During the final hour, the students participated in a debriefing session. They were guided to focus on core systems thinking strategies for successful system performance, including collaboration, innovation, and data-driven decision-making. At the end of the class, the students completed structured open-ended written questionnaires that included three questions: (1) What felt real? (2) What strategies were used during the games? (3) What factors influenced decision-making?

### Data collection and analysis

Data were collected from bar graphs showing team-level quality errors and cost scores, as well as from students’ reflective essays in response to structured open-ended questions. Reflections with missing data were excluded. A hybrid qualitative content analysis was employed to capture both deductive and inductive dimensions of student learning. Strategy-related responses were analysed using directed content analysis, guided by two conceptual frameworks: (1) the core ST strategies embedded in the FNER simulation and (2) the Habits of a Systems Thinker framework, representing foundational HSS competencies (Table 1). This approach enabled the alignment of student behaviours with established theoretical constructions.

**Table 1:** The habits of a systems thinker

Habit	Content
1	Seeks to understand the big picture
2	Observes how elements within systems change over time, generating patterns and trends
3	Recognises that a system’s structure generates its behaviour
4	Identifies the circular nature of complex cause-and-effect relationships
5	Makes meaningful connections within and between systems
6	Changes perspectives to increase understanding
7	Surfaces and tests assumptions
8	Considers an issue fully and resists the urge to come to a quick conclusion
9	Considers how mental models affect current reality and the future
10	Uses understanding of system structure to identify possible leverage actions
11	Considers short-term, long-term, and unintended consequences of actions
12	Pays attention to accumulations and their rates of change
13	Recognises the impact of time delays when exploring cause-and-effect relationships
14	Checks results and changes actions if needed: “successive approximation”

Note. Recreated from “The Habits of a Systems Thinker,” Waters Center for Systems Thinking (CC BY‑NC‑ND 4.0)

Simultaneously, emotional reflections were analysed inductively using conventional content analysis to identify emergent themes without pre-defined categories. Three experienced qualitative researchers (DTC, KHP, and STL) independently reviewed and interpreted the data, identifying patterns of effect and recurring expressions related to the formation of professional identity. The research team engaged in iterative discussions to resolve discrepancies, reach a consensus, and ensure the analytical reliability and validity of the findings.

## Results

### Core strategies used during gameplay in relation to team performance scores

During the FNER gameplay, students applied three core strategies—collaboration, innovation, and data-driven decision-making—at varying levels. Analysis of team performance revealed that Teams 1 and 5 consistently implemented all three strategies, achieving the lowest cost and quality error scores, indicating the highest performance (Table 2). In contrast, Team 4 applied these strategies least effectively and recorded the highest scores, reflecting the lowest performance.

**Table 2:** Core ST strategies used during gameplay in relation to team scores

Core strategy (Total Mentions)	Strategy level	Team Scores (Lower is better = High Performance)				
		Team 1 (30,530)	Team 2 (58,650)	Team 3 (32,920)	Team 4 (78,505)	Team 5 (20,155)
Collaboration (27)	Minimal communication	-	-	-	✓	-
	Communication to convey necessary information	-	✓	-	-	-
	Treating colleagues as customers	-	-	-	-	-
	Joint planning	✓	-	✓	-	✓
	Shared responsibility	✓	-	✓	-	✓
Innovation (18)	Converting unused operating rooms or hallways into emergency rooms	-	-	-	-	-
	Placing two medical staff and two patients in one room	-	-	-	-	✓
	Sharing personnel across team boundaries	✓	✓	✓	-	✓
Data-driven decision-making (21)	Nominal use of available data	-	-	-	-	-
	Leveraging data to drive insights	-	✓	-	-	-
	Using performance indicators to drive improvement	-	-	-	-	-
	Ingrained discipline to seek evidence	✓		✓	-	
	Continuously using data to adapt and grow	-	-	-	-	✓

*“An unexpected accident left a hospital room unusable. We created a new rule that allowed two patients and two medical staff to be in the same room.” Innovation (Team 1)*

*“We looked at the data sheets and discovered that transporting patients by ambulance was the most expensive option while hiring additional staff was more cost-effective. We set goals to reduce transport, minimise waiting times, and efficiently utilise additional staff”. Data-driven Decision-Making (Team 5)*

*“There was a surplus of staff in the surgery room and other departments, while the emergency room was overcrowded. So, our team discussed how to address ER overcrowding and decided to reallocate personnel resources accordingly”. Shared Responsibility (Team 3)*

### Mapping core ST strategies to the habits of a systems thinker

Students’ written essays reflected that the strategies they applied during gameplay were closely aligned with the 14 Habits of a Systems Thinker (Table 3).

**Table 3:** Core ST strategies in relation to the habits of systems thinkers

Team Performance	Core ST Strategies	Representative Quote	Relevant Habits
High (Team 1,3,5)	Collaboration	*“Before starting the game*,* our team collaborated to identify the factors that influence performance. By reviewing the datasheet together*,* we discovered that transferring patients via ambulance incurred the highest cost*,* whereas hiring additional staff was the most cost-effective option. We aligned on shared goals to minimize ambulance transfers*,* reduce patient wait times*,* and efficiently deploy staff when needed.” (Team 1)* *“At the start of the game*,* our team worked together to figure out how we could run the hospital efficiently. We discussed how to allocate resources and set priorities as a group. As time went on*,* we started to notice recurring patterns—every time we delayed hiring staff*,* patient wait times increased and costs spiked. By constantly sharing and analyzing these trends with my teammates*,* I could really feel how our early decisions had ripple effects throughout the system. That’s when we came together again to revise our strategy mid-game and respond in real time. It actually strengthened our teamwork a lot.” (Team 3)* *“At first*,* I thought it was enough to just do my own department’s job well. But as the game progressed*,* I realized that problems in other departments quickly became our problems too. Since we rotated departments every six hours*,* I naturally started to understand the challenges the previous team had faced. That made me pay closer attention to the overall flow of the hospital. Our team kept the hospital’s mission and values in mind*,* and we communicated actively to coordinate things like bed availability and emergency department needs. Through that process*,* I started to feel that we were sharing responsibility—not just for our roles*,* but for the hospital as a whole.” (Team 5)*	► Habit 1. Seeks to understand the big picture ► Habit 2. Observes how elements within systems change over time, generating patterns and trends ► Habit 5. Makes meaningful connections within and between systems
	Innovation	*“At one point*,* the ER was clearly understaffed*,* patients were flooding in*,* and unexpected problems kept popping up. Honestly*,* part of me just wanted to follow the standard rules and hope things would get better. But as a team*,* we paused and started to question our assumptions. Instead of treating each department as its own island*,* we asked*,* ‘What if we could move staff across team boundaries?’ and ‘What if two patients and two medical staff shared a room?’ That shift in thinking helped us realize we’d been stuck in a rigid mindset. Once we let go of that and started thinking more flexibly*,* we came up with creative and actually workable solutions to deal with the crisis.” (Team 3)* *“Due to an unexpected event*,* the hospital room became unusable. A new rule was established to allow multiple people to enter one room*,* with two patients and two medical staff members being placed together.” (Team 1)*	► Habit 6. Changes perspectives to increase understanding ► Habit 8. Considers an issue fully and resists the urge to come to a quick conclusion ► Habit 9. Considers how mental models affect current reality and the future
	Data-driven Decision-making	*“By reviewing the datasheet*,* we realized that transferring patients by ambulance was the most expensive option while hiring additional staff was the most cost-effective. We set goals to reduce ambulance use*,* minimize wait times*,* and efficiently utilize added personnel.” (Team 5)* *“Since it takes one hour for newly hired staff to become available*,* we anticipated delays and hired staff in advance to avoid treatment delays.” (Team 3)* *“At first*,* I underestimated how fast small issues could escalate. But by monitoring real-time data like patient numbers and staffing shortages*,* I saw how delays and missed decisions caused bigger problems. We stopped relying on fixed plans and adjusted our actions based on the data—like hiring staff early and avoiding unnecessary transfers. I learned that data-driven decisions and flexibility are key to managing both immediate challenges and overall system flow.” (Team 1)*	► Habit 11. Considers short-term, long-term and unintended consequences of actions ► Habit 12. Pays attention to accumulations and their rates of change ► Habit 13. Recognises the impact of time delays when exploring cause-and-effect relationships ►Habit 14. Checks results and changes actions if needed: “successive approximation”
Mid (Team 2)	Collaboration	*“At first*,* I just shared what was happening in my room*,* but I soon realized that one department’s problem affected the ER*,* which then impacted other rooms. I saw how the system’s structure connected everything and created recurring issues.” (Team 2)*	► Habit 3. Recognises that a system’s structure generates its behaviour ► Habit 4. Identifies the circular nature of complex cause-and-effect relationships
	Innovation	*“Even though we kept hiring more staff*,* patients were still piling up in the ER*,* and I assumed it was because there weren’t enough beds. But after reviewing the cost and quality performance charts*,* I realized the real issue was wait time—and that we could bring in staff from other departments. It hit me that I’d been thinking inside a box the whole time. I wish I had shifted my perspective sooner; we might have made better decisions earlier.”(Team 2)*	► Habit 6. Changes perspectives to increase understanding ► Habit 7. Surfaces and tests assumptions ► Habit 8. Considers an issue fully and resists the urge to come to a quick conclusion
	Data-driven Decision-making	*“Midway through the game*,* we revisited the data and realized that the cost of having a patient wait was about the same as hiring four additional staff members. We also saw clearly that transferring patients to other hospitals had the most negative impact on patient safety and quality. That’s when we changed our strategy. Looking back*,* I wish we had questioned our assumptions and paid closer attention to the data earlier—our decisions might have been better from the start.” (Team 2)*	► Habit 6. Changes perspectives to increase understanding ► Habit 7. Surfaces and tests assumptions
Low (Team 4)	Collaboration	*“Each department did check how many patients were admitted and discharged and whether there were any requests*,* but we didn’t really communicate that information with each other. Everyone was focused on their own room*,* and we missed the bigger picture or what other teams might have needed. Looking back*,* I think if we had communicated and collaborated more*,* the hospital could have run much more smoothly. That’s something I regret.”(Team 4)*	-

To enhance reader understanding, we associated each habit with a key concept and referenced both the concept and number in the text. Habits related to understanding situations and designing strategies—such as seeing the big picture (Habit 1), recognising patterns over time (Habit 2), and identifying system structure (Habit 3)—were more frequently observed in high-performing teams. Habits involving the exploration of alternatives and challenging mental models—such as changing perspectives (Habit 6), surfacing assumptions (Habit 7), and resisting premature conclusions (Habit 8)—appeared during innovative thinking. Decision-making was guided by habits focused on outcomes and feedback loops, such as considering long-term consequences (Habit 11), tracking accumulations (Habit 12), recognising time delays in cause-and-effect (Habit 13) and adapting based on results (Habit 14). Teams that applied all three core ST strategies—collaboration, innovation, and data-driven decision-making—demonstrated a dynamic integration of all 14 habits, whereas those who failed to employ these strategies showed limited or no use of the habits.

### Collaboration

Collaboration varied across teams. High-performing teams (Teams 1, 3, and 5) shared goals, analysed data collaboratively, and coordinated interdepartmental actions effectively. These students demonstrated ST habits such as seeing the big picture (Habit 1), recognising changing patterns (Habit 2), and making meaningful connections (Habit 5). One student wrote:

*“Before starting the game, our team collaborated to identify the factors that influence performance. By reviewing the datasheet together, we discovered that transferring patients via ambulance incurred the highest cost, whereas hiring additional staff was the most cost-effective option. We aligned on shared goals to minimize ambulance transfers, reduce patient wait times, and efficiently deploy staff when needed.” (Team 1)*

Lower-performing teams, such as Team 4, demonstrated minimal collaboration and lacked a shared vision, resulting in siloed decision-making and reduced performance.

### Innovation

Students devised creative yet sometimes imperfect solutions, such as placing two patients and two staff in a single room or reallocating staff across departments. These behaviours reflected habits like changing perspectives (Habit 6), surfacing assumptions (Habit 7), and resisting premature conclusions (Habit 8). For example:

*“Due to an unexpected event, the hospital room became unusable. A new rule was established to allow multiple people to enter one room, with two patients and two medical staff members being placed together.” (Team 1)*

Even when not fully effective, such innovations reflected a shift from conventional thinking, emphasising the game’s ability to provoke adaptive problem-solving.

### Data-driven decision-making

Many students relied on data to improve their performance, referencing real-time charts to adjust staffing and patient flow. High-performing teams (Teams 1, 3, and 5) illustrated habits such as evaluating results (Habit 11), tracking accumulations (Habit 12), recognizing time delays (Habit 13), and adapting based on outcomes (Habit 14). One student wrote:

*“By reviewing the datasheet, we realized that transferring patients by ambulance was the most expensive option while hiring additional staff was the most cost-effective. We set goals to reduce ambulance use, minimize wait times, and efficiently utilize added personnel.” (Team 5)*

*“Since it takes one hour for newly hired staff to become available, we anticipated delays and hired staff in advance to avoid treatment delays.” (Team 3)*

Students also demonstrated an understanding of system structure (Habit 3) and recognition of feedback loops (Habit 4) when discussing the broader impact of departmental problems on the entire hospital system. Team performance reflected the degree to which these habits were employed. High-performing teams (Teams 1, 3, and 5) consistently and comprehensively applied the habits, showing precise alignment between strategies and outcomes. Team 2, with moderate performance, showed emerging awareness and partial application of the habits, albeit inconsistently. In contrast, low-performing teams (Team 4) lacked collaboration and demonstrated minimal application of systems thinking habits, resulting in reactive decisions and suboptimal outcomes. Notably, Habits 1 (seeing the big picture), 6 (shifting perspectives), and 11 (considering long-term consequences) were rarely observed in low-performing teams.

### Participants’ emotions and insights regarding the FNER game

Students’ reflections on the FNER simulation were categorized into five key themes that captured their emotional responses and learning insights. Table 4 provides representative quotations for each theme. These themes highlight how the game fostered deep reflection on real-world healthcare challenges and systems thinking.

**Table 4:** Five thematic reflections on emotions and learning from the FNER game

Theme	Representative quotation
Importance of collaboration and teamwork	*“While playing this game*,* I realised the importance of teamwork. […] Even when we held different viewpoints*,* we discussed them and reached an agreement without significant conflict. As a result*,* the game progressed smoothly.” (MS 1_15)* *“Although each team member had a different role*,* I focused on understanding the overall situation of the hospital. I continuously monitored staff allocation*,* bed availability*,* and the need for additional personnel*,* emphasising running the hospital collaboratively rather than concentrating only on a single department.” (MS 1_4)*
Value-driven decision-making	*“The presence of patients on standby strongly influenced my decisions. To reduce waiting time*,* we added medical staff*,* accepting higher initial costs in favour of improved patient care in the long term.” (MS 1_20)* *“Although hiring more doctors increased costs*,* our team prioritised stable hospital operations and patient care over cost reduction alone.” (MS 1_10)*
Innovation and change management	*“To ensure continuous care for patients*,* we created a flexible environment where treatment could be provided even in the emergency room’s waiting area.” (MS 1_14)* *“As unexpected situations kept occurring*,* I realised that we couldn’t solve the problems using the existing methods alone. So*,* our team created new rules and changed the way the hospital operated in an effort to turn the crisis into an opportunity.” (MS 1_5)*
Patient-centred care	*“While considering how to allocate limited resources*,* I deeply realised that what may seem like just a score in the game could*,* in reality*,* be connected to a person’s life.” (MS 1_7)* *“We did not hesitate to add staff*,* if necessary. We aimed to avoid transferring emergency patients to other hospitals.” (MS 1_18)*
Indirect hospital system experience	*“I realised how important leadership is in hospital management. I regretted not being able to help emergency patients due to financial concerns*,* lack of decision-making knowledge*,* and unclear demands. Observing other teams made me realise the significance of having a clear vision and goals in an organisation.” (MS 1_19)* *“I learned how a real hospital operates through the cooperation of various departments. I felt as if I were working in a real hospital*,* trying to find the best ways to treat my patients.” (MS 1_3)* *“I could feel the anxiety that both patients and doctors experience when there is a shortage of beds. Often*,* events occur unpredictably*,* and the ability to respond promptly was crucial.” (MS 1_24)*

### Importance of collaboration and teamwork

The first theme, the importance of collaboration and teamwork, emerged prominently in student reflections. Many participants emphasized that, prior to the start of the game, their teams collaborated closely to understand the overall hospital system and to devise the most effective operational strategies. This early effort to establish a shared understanding of the system’s structure and interdependencies contributed to a smooth gameplay experience. Students recognized that such proactive coordination mirrors the strategic planning and interdepartmental alignment required for effective hospital management.

*“While playing this game*,* I realised the importance of teamwork. […] Even when we held different viewpoints*,* we discussed them and reached an agreement without significant conflict. As a result*,* the game progressed smoothly.” (MS 1_15)*

### Value-driven decision-making

With regard to value-driven decision-making, students underscored the ethical responsibility to prioritize patient lives over financial considerations. Several participants referenced the social reality of emergency department overcrowding—commonly described as “ED rotation” or “patient ping-pong”—as a critical challenge. They reflected that the simulation helped them internalize the need for structural reform and staffing innovation to protect patient safety. The activity reinforced the principle that healthcare decisions should fundamentally be guided by the value of human life.

*“The presence of patients on standby strongly influenced my decisions. To reduce waiting time, we added medical staff, accepting higher initial costs in favour of improved patient care in the long term.” (MS 1_20)*

### Innovation and change management

The importance of innovation and change management also surfaced as a recurring theme.

Students noted that when healthcare systems become entrenched in routine practices, it becomes difficult to generate new solutions. However, the simulation encouraged them to adopt fresh perspectives and to embrace innovative thinking, particularly when faced with complex and dynamic challenges. They came to understand that reframing problems and adapting existing rules can be crucial for achieving sustainable improvements.

*“To ensure continuous care for patients, we created a flexible environment where treatment could be provided even in the emergency room’s waiting area.” (MS 1_14)*

### Patient-centred care

The fourth theme centered on patient-centred care. Students reported that the game effectively illustrated the need to deliver timely and appropriate care and highlighted the crucial role of hospital systems in supporting this goal. Through gameplay, they engaged in balancing decisions about limited resources while maintaining a consistent focus on improving patient outcomes. This experience heightened their awareness of the organizational commitment required to center care around patient needs.

*“While considering how to allocate limited resources, I deeply realised that what may seem like just a score in the game could, in reality, be connected to a person’s life.” (MS 1_7)*

### Indirect hospital system experience

Students reflected on their indirect experiences with the hospital system, playing the game with a sense of urgency while dealing with ambiguity. They reported that the simulated environment mirrored natural hospital settings where health professionals often face the unknown. Additionally, the students shared that simulation gameplay elicits emotions commonly experienced by leaders and managers in natural healthcare settings.

Students reflected on the feelings of reality and urgency that the simulated hospital evoked in them during gameplay. They felt like the medical staff were working in a real setting and had to deal with healthcare problems and management issues.

*“I realised how important leadership is in hospital management. I regretted not being able to help emergency patients due to financial concerns*,* lack of decision-making knowledge*,* and unclear demands. Observing other teams made me realise the significance of having a clear vision and goals in an organisation.” (MS 1_19)*.

## Discussion

The findings of this study show that the FNER simulation game is an effective educational tool for teaching the concepts and principles of ST, as outlined in the HSS, to medical students before they enter clinical clerkships. It allows them to reflect on the 14 habits of systems thinkers. This supports the outcomes of previous studies suggesting that experiential learning with the FNER simulation game helps students’ ST, which is an important element in this game. Previous studies by Thornton Bacon [49], Sanko and colleagues [38, 40] and Fusco [35, 36] have focused on investigating the improvement in the student’s ST through quantitative data analysis. In each study, the authors found a statistically significant increase in the students’ systems thinking scores after engaging in FNER games. While previous quantitative studies merely demonstrated an increase in ST scores, our study provides qualitative evidence that ST was actively learned through collaboration, innovation, and data-driven decision-making. Specifically, our analysis showed that the application of the three core systems thinking (ST) strategies—collaboration, innovation, and data-driven decision-making—was strongly aligned with specific Habits of a Systems Thinker. Collaboration was most closely associated with Habit 1 (Seeks to understand the big picture), innovation with Habit 6 (Changes perspectives to increase understanding), and data-driven decision-making with Habit 11 (Considers short-term, long-term, and unintended consequences of actions). Furthermore, the presence or absence of these habits varied depending on the strategic level demonstrated by each team, which appeared to be shaped by how individual mental models influenced collective team decision-making. These findings provide a nuanced understanding of how systems thinking manifests not only at the individual level but also through team-level dynamics during gameplay. Moreover, beyond ST, preclinical students were able to indirectly engage with the pressures of hospital operations and patient safety issues. The study also confirmed that teams with higher performance scores were more likely to apply ST effectively. Our data contribute to the existing literature by examining qualitative data on students’ reflections and providing valuable insights into their experiences. Our findings support the use of structured gaming as a valuable strategy for enhancing students’ exposure to ST, enabling them to apply ST concepts in simulated environments [36, 38, 40, 49, 51]. This also supports prior research suggesting that ST education in healthcare should go beyond classroom-based knowledge acquisition and emphasise learner-centred approaches that provide experiential, practice-based learning opportunities [1, 7].

The FNER game promotes empathy and awareness among future doctors. Students reflected on resource scarcity, bed shortages, and delayed decisions—not as abstract inefficiencies but as tangible risks to patient safety. For example, students noted that avoiding patient transfers was not just a cost-saving measure but a patient-centred decision aligned with ethical care. For healthcare providers, the FNER game emphasises interdepartmental communication and coordination. Students experienced the challenges of managing shared resources and learned to prioritise collective outcomes over siloed responsibilities. This aligns with research on the importance of team-based care in complex healthcare environments [5, 46]. The findings demonstrate the potential of simulation-based education in preparing medical students to operate effectively in system-based healthcare environments. Especially in the Korean context, where systemic inefficiencies and physician dissatisfaction are rising, our results argue for embedding ST education within broader reform efforts. Simulations like FNER can expose future clinicians to systemic challenges before they enter clinical training, potentially fostering system-aware leadership. Our results support the integration of HSS and ST into preclinical curricula. The use of reflection-based learning, grounded in real-time simulation, allowed students to internalise the 14 habits of systems thinkers. Unlike didactic instruction, simulation games prompt emotional engagement, critical decision-making, and habit formation.

Our findings contribute to the literature by illustrating how experiential simulation can bridge the gap between theory and practice. While previous research has called for a shift from knowledge-based to competency-based medical education [1, 7, 46], our study operationalises this shift by linking gameplay strategies to ST habits and connecting these with authentic hospital management challenges. Moreover, the emotional responses observed—anxiety, urgency, and frustration—mirror real clinical pressures and validate the simulation’s fidelity. This supports the perspectives of Kolb [50] and Eraut [51], who emphasise the importance of emotional and social dimensions in professional learning.

South Korea faces unique structural challenges in implementing HSS education. Despite a government-led national health insurance system, medical students often bear the financial burden of their training and lack exposure to healthcare systems education. This disconnectedness between individual-level training and public-level healthcare infrastructure contributes to ongoing tensions between physicians and policymakers. We propose that Korea’s Ministry of Education and the Ministry of Health and Welfare collaborate to develop and fund integrated ST and HSS curricula in undergraduate medical education. Preparing future physicians as both clinicians and system-aware leaders requires institutionalising experiential learning approaches such as the FNER simulation game. Doing so can enhance quality, equity, and innovation in healthcare delivery.

Nonetheless, several limitations should be acknowledged in this study. First, the students who participated in this research chose the course ‘Leadership and Systems Thinking’ as an elective course, thereby raising the possibility of selection bias; moreover, our participants may not comprise a random sampling of students. Second, as the study involved only medical students, future research should explore interprofessional education by including students from various healthcare disciplines to gain broader perspectives. This highlights the need for designing and evaluating systematic curricula aimed at enhancing systems thinking competencies. Moreover, structured and professional faculty development should be prioritised to support experiential learning through simulation games and effective debriefing sessions.

Additionally, we conducted this study in one medical school with a relatively small sample size, which may affect the generalisability of the findings. However, as this is a qualitative study, the focus was on obtaining in-depth insights and understanding the students’ reflections on their experiences. Although the findings may not be widely generalisable, our research provides a deeper understanding and insight into the impact of experiential learning activities using simulation games and thereby contributes to current literature. Finally, the study design involved only a single gameplay session, which may have limited data depth. Monitoring participants over multiple sessions could offer richer data on the development of systems thinking skills and provide a clearer picture of how simulation-based learning influences both performance outcomes and student evaluations over time.

## Conclusions

This study highlights the effectiveness of using the FNER simulation game to foster ST and HSS among preclinical medical students. Through immersive gameplay, students engaged with the 14 Habits of a Systems Thinker and practised core ST strategies—collaboration, innovation, and data-driven decision-making—while navigating hospital-level challenges.

Unlike prior studies focused on knowledge gains, our qualitative approach revealed how experiential learning supports the formation of early professional identity as systems-aware clinicians. Integrating structured simulations and debriefings into the curriculum may offer a practical and scalable approach to embedding ST and HSS in undergraduate medical education, with implications for enhancing patient care and health system performance.

## Data Availability

The data that support the findings of this study are available on request from the corresponding author, [STL, sangmilee@korea.ac.kr].

## Abbreviations

FNER: Friday night at the ER
HSS: Health systems science
ST: Systems thinking
